# The effects of self-esteem and parental phubbing on adolescent internet addiction and the moderating role of the classroom environment: a hierarchical linear model analysis

**DOI:** 10.1186/s12888-023-05489-y

**Published:** 2024-01-19

**Authors:** Bibing Dai, Yan Lin, Xinyi Lai, Jiankang He, Mingxuan Du, Xiaowen Hou, Guohua Zhang

**Affiliations:** 1 Department of Psychiatry and Psychology, School of Basic Medical Sciences, Tianjin Medical University, Tianjin, China; 2 Department of Psychology, School of Mental Health, Wenzhou Medical University, Wenzhou, China; 3 Zhejiang Provincial Clinical Research Center for Mental Disorders, The Affiliated Wenzhou Kangning Hospital, Wenzhou Medical University, Wenzhou, China

**Keywords:** Internet addiction, Self-esteem, Parental phubbing, Classroom environment, Hierarchical linear model, Chinese adolescents

## Abstract

**Background:**

With the advent of the new media era, the understanding of adolescent internet addiction needs to be enriched. It is also necessary to distinguish the related factors of adolescent internet addiction at different levels to clarify the mechanisms of this phenomenon.

**Methods:**

This study used hierarchical linear model analysis to explore the effects of student-level factors and school-level factors on adolescent internet addiction, along with cross-level moderating effects. A total of 1,912 students between the 4th and 8th grades in China participated in the study. Participants completed the Self-Esteem Scale, Parents Phubbing Scale, Classroom Environment Scale, and the Diagnostic Questionnaire of Internet Addiction.

**Results:**

Correlational analyses revealed that internet addiction was found to be negatively correlated with both self-esteem and the teacher-student relationship (*p* < 0.01), while father phubbing, mother phubbing, and learning burden were shown to positively correlate with internet addiction (*p* < 0.01). Hierarchical linear model analysis suggested that student-level variables, including self-esteem, and mother phubbing, were significant predictors of internet addiction (*β* = −0.077, *p* < 0.001 and *β* = 0.028, *p* < 0.01, respectively). At the school level, learning burden significantly and negatively predicted internet addiction (*β* = 0.073, *p* < 0.05). Furthermore, the relationship between self-esteem and internet addiction was significantly moderated by learning burden (*β* = −0.007, *p* < 0.05). Meanwhile, the teacher-student relationship also had a significant moderating effect on the association between mother phubbing and internet addiction (*β* = −0.005, *p* < 0.01).

**Conclusions:**

This study revealed the relationships between self-esteem, parental phubbing, and classroom environment with adolescent internet addiction, and these findings could provide insights into reducing adolescent internet addiction from the perspective of individuals, families, and schools.

## Introduction

In the era of developed internet and network technology, the internet is used for communication, education, and entertainment, having become a necessary part of people’s daily lives. However, with the advantages of convenience and multi-functions, the challenges that individuals can easily become addicted to online activities are emerging. One of the most harmful instances of this phenomenon is often known as internet addiction (IA). According to the diagnostic criteria of pathological gambling in the Diagnostic and Statistical Manual of Mental Disorders, 4th Edition (DSM-IV), Young defined IA as an impulsive control disorder characterized by an inability to inhibit internet surfing drives that damages major life domains and social functions [1]. Because of the topic’s complex nature, related terms such as “internet behavior dependence” [2] and “pathological internet use” [3] are usually regarded as synonyms for IA.

Adolescents are highly predisposed to IA. Nowadays, it is becoming more common and inevitable for young people to use the internet for communication, leisure and recreation, and completing learning tasks [4, 5]. At the same time, adolescents are in a psychological period in which they desire independence and autonomy, while their self-control and self-regulation are poorer than those of adults, which makes them more prone to IA [6]. The prevalence of IA among adolescents in China is disquieting, ranging from 8.8 to 12.2% [7, 8], and the prevalence rates might further increase in the coming years. Besides, excessive internet use may also increase the risk of negative psychosomatic symptoms and conduct problems, such as anxiety, depression, academic difficulties, poor interpersonal relationships, and even a high risk of suicide [9–11]. Therefore, many scholars have strongly suggested the classification of IA as an adolescent risk behavior [12], and that specific attention needs to be paid to adolescent IA in China [13].

According to Ecological Systems Theory [14], individual behavior is a result of the interaction between humans and their environment, which means that, in addition to the individual’s development, the ecological environment in which an individual lives also has a significant impact on individual development. For adolescents, the family and the school are the earliest and closest environmental systems, and their key members (i.e., parents and teachers) can help shape their behavior through communication and interaction. However, to our knowledge, current studies are more based on individual characteristics, family components, school climate, and other single factors or two factors to explore. There is an urgent need for the measurement and screening of data from joint systems, including individual-level and school-level data, to ultimately promote the involvement of multi-level stakeholders [15]. Therefore, our research used a large sample of Chinese adolescents to analyze the associations of individual- and school-level factors with adolescent IA, as well as any interactions between these factors.

### Self-esteem and adolescent internet addiction

Self-esteem is an individual psychological trait that can be described as a self-evaluation of one’s image and part of the self-concept made up of the positive terms that people use to describe themselves [16]. According to Rosenberg [17], self-esteem has always been closely linked to physical and psychological factors and behavior problems. Similarly, self-esteem may also have a significant impact on adolescent IA [18]. Specifically, teenagers who have low self-esteem in general lack self-confidence and avoid interactions with others in the real world. They prefer communicating online to keep their confidence intact, thus deepening their dependence on the internet [19]. Many studies have shown that self-esteem is negatively correlated with IA, but the association between them may be moderated by other factors, and this is an area that still needs further clarification. For example, one review suggested that variables such as gender and age may affect the strength and even the nature of this relationship [20]. Therefore, it is necessary to further investigate the relationship and underlying mechanisms between them to deploy the favorable role of self-esteem.

### Parental phubbing and adolescent internet addiction

Family and parent-child relationships can also provide an important contribution to understanding the pathogenesis of adolescent IA, especially at a pubescent age [5, 21]. As an emerging phenomenon, parental phubbing has also attracted attention and has been found to positively predict adolescent IA in some empirical studies. Parental phubbing refers to a social behavior whereby parents neglect their children, focusing on their mobile phones instead during parent-child interactions [22–24], which emphasizes the children’s negative experience of being ignored [25]. A study of primary school students in China showed that children felt discontented with their parents’ phubbing because it conveyed a signal of alienation from parents, which aggravated children’s dependence on the internet [26]. Geng et al. verified that adolescents who perceived more parental phubbing in the early interaction will experience more feelings of loneliness and eventually become addicted to the internet [27]. Some scholars considered working from home would increase the time of parent-child interaction during the COVID-19 pandemic, and their study design demonstrated that frequent parent phubbing would mislead adolescents to think that phubbing is socially accepted, which would induce adolescent IA [28]. Given the results of previous research, parental phubbing may increase the risk of adolescent IA, and further testing for the mechanism underlying this relationship is needed. However, parental phubbing was mostly measured as a whole in previous studies, rather than father’s and mother’s phubbing [24, 29]. Family system theory points out that the family is a hierarchical system in which the interactions between each member are complex, and there are mutual influences and interactions between them [30]. Meanwhile, in consideration of the different family functions of the family members, the mother often plays a more important role in nurturing the child’s emotions, while the father is more about guiding social rules and contact [27, 31]. It is necessary to explore the relationships between fathers’ and mothers’ phubbing and adolescent IA separately.

### Classroom environment and adolescent internet addiction

Classroom environment is also a significant factor associated with adolescent IA [32, 33]. The classroom environment refers to a relatively stable environment state of class that includes all the student’s perceptions of their class and which influences the students differently at the same time [34]. In other words, it is a multi-level construct that contains an overall organizational structure, norms, and culture and can represent the interaction quality of all the school members [35]. As its important components, learning burden and teacher-student relationships have been examined by scholars in the field of IA.

Because of the particular cultural and educational background that requires taking a highly competitive college entrance examination in China, Chinese teenagers spend most of their time in school, striving for better academic performance, which means that most of them bear a very large learning burden [36]. Prior empirical studies have indicated that academic pressure is one of the main sources of personal life pressure for adolescents, and adolescents that fail to cope with their negative emotions (e.g., depression, anxiety and feeling of inferiority) and cognition (e.g., perception, attention, memory and intelligence) caused by academic stress often develop IA [36, 37]. Thus, there is an urgent need to further clarify the relationship between learning burden and IA for adolescents, especially based on the perspective of the classroom environment.

Along with parents, teachers are key figures who guide and support the growth of teenagers, and the adults who accompany teenagers for a long period [38]. Attachment theory points out that the relationship between teachers and students is a continuation of parent-child attachment relationships [39]. Empirical evidence has shown that the more intimate the relationship that adolescents establish with their teachers, the less likely they are to engage in excessive internet use [40]. Other studies with a sample of Chinese middle school students have also confirmed the protective effect of teacher-student relationships on IA [36, 41]. Therefore, it is essential to investigate the relationship between teacher-student relationship and IA for the prevention and intervention of IA.

In addition, research on the conduct of moderators across the individual-level is still emerging. Jia and Li insisted that students are more likely to experience differences in the way they are impacted by the same adverse event in the classroom [40]. Their finding showed that harmonious teacher-student relationships moderated the negative impact of peer victimization on IA, which revealed that the classroom environment is a more complex property in reality that various parts of factors may perform different functions. Meanwhile, Chang and Kim also identified many two-level interaction effects between individual variables (including gender, delinquency, and self-esteem) and school climate on internet gaming addiction [18]. Thus, based on previous findings, it was necessary for this study to analyze the specific effects of cross-level (e.g., student and school level) interactions on adolescent IA.

### The current study

Adolescent IA has earned substantial attention from scholars, and there are abundant research achievements in related fields. However, previous studies have also demonstrated that there existed remarkably different influences between individual factors and external environmental factors on adolescent IA [42], but few studies examined specific joint mechanisms of the two aspects of factors and seldom analyzed both the family environmental system and school environmental system [36]. The interaction between the environmental system and adolescent IA may provide a special perspective to complement existing knowledge. Furthermore, it is also a meaningful design to divide parental phubbing into mother’s and father’s phubbing. As we knew that the different divisions of family labor cannot be ignored [43].

As mentioned above, diverse findings in these studies were likely caused by their discrepant inclusion of influencing factors, lack of group perspectives, and different cultural contexts, which may influence relevant associations with adolescent IA. To date, most of the existing research does not provide adequate evidence to demonstrate the combined effect of individual and environmental factors on adolescent IA, since the classroom environment was often analyzed only as a student-level variable rather than as a group factor [44].

Therefore, to better understand the various factors and complex mechanisms acting on adolescent IA, this study was designed to construct a two-level linear model with a large student sample located in mainland China. Accordingly, we aimed to identify some of the student-related factors (self-esteem and parental phubbing) and school-related factors (learning burden and teacher-student relationship) linked to adolescent IA via multilevel analysis. Furthermore, we aimed to examine the moderating effects underlying these relationships via cross-level analysis.

## Hypotheses

**Fig. 1:**
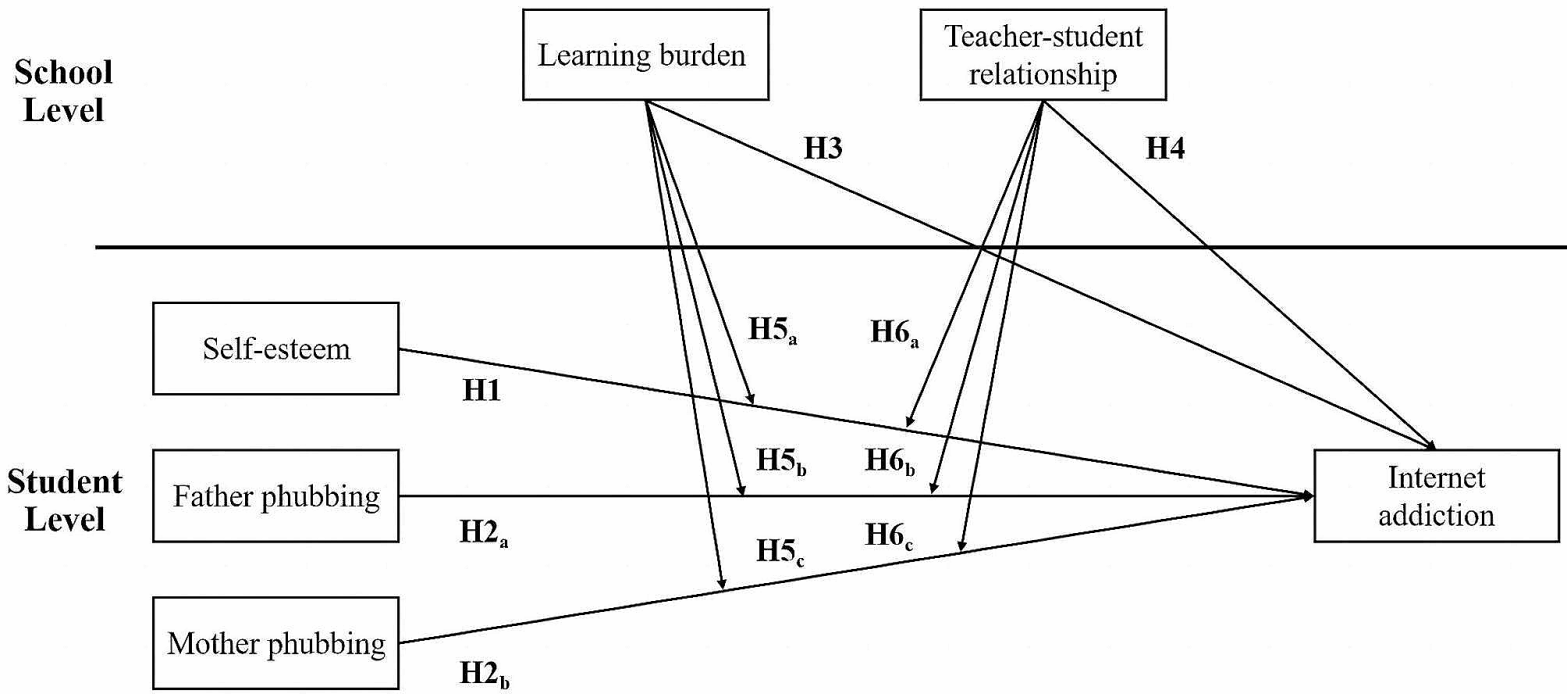
The conceptual framework of the study

The current study aimed to contribute to a more comprehensive understanding of IA among Chinese adolescents and help relevant departments develop education programs to prevent IA. The hypotheses that we proposed are as below (see Fig. 1). It should be noted that due to the complexity of cross-level interactions (i.e., the interactions between the school-level variables and the student-level variables may exist many possibilities), the present study treats them as open questions for which no specific hypotheses are evident.

H1: At the student level (level 1), self-esteem would negatively and significantly predict adolescent IA.

H2: At the student level (level 1), father phubbing (H2_a_) and mother phubbing (H2_b_) would positively and significantly predict adolescent IA.

H3: At the school level (level 2), the learning burden would positively and significantly predict adolescent IA.

H4: At the school level (level 2), the teacher-student relationship would negatively and significantly predict adolescent IA.

H5: The interactions between learning burden and variables at the student level, including self-esteem (H5_a_), father phubbing (H5_b_), and mother phubbing (H5_c_), would be significantly associated with adolescent IA.

H6: The interactions between the teacher-student relationship and variables at the student level, including self-esteem (H6_a_), father phubbing (H6_b_), and mother phubbing (H6_c_), would be significantly linked to adolescent IA.

## Method

### Participants and procedure

To ensure the inclusion of a range of socioeconomic backgrounds, we gathered students through convenience sampling across mainland China in East China (e.g., Jiangsu and Zhejiang Province), South China (e.g., Guangdong and Fujian Province), and Central China (e.g., Henan Province) from November 2021 to February 2022. We obtained informed consent from the school principal, students, and their parents before our investigation. We asked teachers in sample schools to distribute paper-and-pencil questionnaires to students after the training. Participating students completed this anonymous questionnaire during a self-study session and were encouraged to ask questions if anything was unclear. A total of 2,082 students from grade four to grade eight participated in the current study, and 170 (8.2%) were removed because the unfilled part of their questionnaire exceeded 20%. The remaining 1,912 students from 50 classes contained 1,004 boys (52.5%) and 908 girls (47.5%); 1,603 (83.8%) were from urban regions, while 309 (16.2%) were from rural regions. Among them, the mean age was 11.03 (*SD* = 1.13) years, and the number of students in grades four, five, six, seven, and eight accounted for 28.0%, 25.9%, 36.6%, 4.6%, and 5.0%, of the total, respectively. The present survey was also approved by the corresponding author’s university institutional ethics board (KNLL-20,211,011,002).

### Measures

Three measures served as independent variables at the student level in the current study, including socio-demographic variables (i.e., age and gender), self-esteem, and parental phubbing. Besides, the independent variables at the school level were mainly obtained from the classroom environment scale, which contained the learning burden and the teacher-student relationship. The dependent variable was adolescent IA.

#### Self-esteem scale

The 10-item Rosenberg Self-Esteem Scale was used to assess one’s character of self-esteem [45]. Items were rated on a 4-point Likert scale ranging from 1 (*strongly disagree*) to 4 (*strongly agree*). A sample item was “I feel that I am a person of worth, at least on an equal plane with others”. Higher scores reflected a higher level of self-esteem. The Cronbach’s *α* of the scale was 0.849 in this study.

#### Parents phubbing scale

In the current study, a revised version of the 9-item Parents Phubbing Scale was used to measure students’ perceived parental phubbing behavior [46]. Items were scored on a 5-point Likert scale ranging from 1 (*never*) to 5 (*all the time*), and the phubbing behavior of both parents was collected (e.g., “My parent glances at his/her cell phone when talking to me”). Higher scores indicated greater degrees of paternal/maternal phubbing behavior. In this study, the Cronbach’s *α* of paternal and maternal phubbing behavior were 0.858 and 0.851, respectively.

#### Classroom environment scale

Two dimensions of the “My Class” Questionnaire, including “learning burden” and “teacher-student relationship” were selected to evaluate students’ classroom environment in this study [47]. The sub-scales comprised 15 items rated on a 5-point Likert scale ranging from 1 (*never*) to 5 (*all the time*). Sample items were “Students feel a lot of pressure to study” and “The headteacher genuinely cares about classmates.” Higher scores of sub-scales signified the heavier learning burden, and the better relationships between teachers and students, respectively. In this study, the Cronbach’s *α* of the two sub-scales were 0.774 and 0.912.

#### The diagnostic questionnaire of internet addiction

The measurement of adolescent generalized IA was conducted using Young’s 8-item Diagnostic Questionnaire of Internet Addiction (IAD-DQ) which was based on the criteria for pathological gambling in DSM-IV [1]. The 8 items were rated 0 (*No*) or 1 (*Yes*), and higher scores indicated a more serious degree of one’s IA (e.g., “Do you use the internet as a way of escaping from problems or of relieving a dysphoric mood?”). The Chinese version has been tested for good reliability [48], and Cronbachʼs *α* of IAD-DQ was 0.752 in our study.

### Data analysis

Descriptive statistics, which included means and standard deviations (SD), were calculated, and a correlation analysis was also conducted to explore the associations among variables.

Hierarchical Linear Modeling (HLM), also called muti-level modeling, is a cross-level analysis that intuitively presents the influence of independent variables on dependent variables [49]. It is especially suitable for nested data structures, such as students nested within a class or a school, in educational research [50]. Considering the collected data was characteristic of the hierarchical structure [51], a two-level multilevel analysis was adopted in the current study. Therefore, we utilized HLM 6.08 multilevel modeling software for data analysis in which the student-level variables (i.e., self-esteem, father and mother phubbing) are based on individual students’ responses, while school-level variables are based on aggregated responses of students within a class (i.e., class-aggregated learning burden and class-aggregated teacher-student relationships), and the specific steps were as follows:

#### Step 1

Null model (Model 1). To identify significant differences in students’ IA between classes and whether HLM is adequate and suitable for our research, we calculated a null model without predictive variables at all levels, which only includes the dependent variable, IA. Meanwhile, we examined the intra-class correlation coefficients (ICC), ICC_1_ and ICC_2_. ICC_1_ compares the between-group variance to the within-group variance to indicate the portion of the variance in individual responses that is accounted for by between-group differences [52]. ICC_2_ reveals the reliability of the mean of a level 2 variable [53, 54].

#### Step 2

Random coefficients regression model (Model 2). To measure the effects of student-level factors (i.e., self-esteem, father, and mother phubbing) on IA, all predictive variables at the student level were brought into the level one equation, and some of the demographic variables (i.e., age and gender) were controlled as they might particularly salient for IA.

#### Step 3

Full model (Model 3). All class-level predictive variables were added to the level two equation of the model from step 2, to determine the effects of school-level predictors (i.e., learning burden and teacher-student relationship) on adolescent IA, as well as the cross-level interaction effects between student- and school-level predictors.

## Results

### Descriptive statistics and correlation analysis

Table 1 gives the correlation matrix, means, and standard deviations for the variables. IA was found to be negatively correlated with both self-esteem and the teacher-student relationship, while father phubbing, mother phubbing, and learning burden were shown to positively correlate with IA.

**Table 1:** The correlations among variables, means, and standard deviation

Variables	1	2	3	4	5	6
1. Internet addiction	1					
2. Self-esteem	−0.315**	1				
3. Father phubbing	0.210**	−0.193**	1			
4. Mother phubbing	0.241**	−0.204**	0.708**	1		
5. Learning burden	0.242**	−0.184**	0.222**	0.237**	1	
6. Teacher-student relationship	−0.226**	0.237**	−0.135**	−0.146**	−0.387**	1
*M*	1.196	30.263	20.760	19.585	17.007	33.400
*SD*	1.703	5.733	7.891	7.458	2.805	3.789

*Note*: * *p* < 0.05, ** *p* < 0.01

### HLM analysis

#### Results for the null model

The Model 1 is as follows:

Level 1 (student-level):

$${Y_{ij}}\,=\,{\beta _{0j}}\,+\,{\varepsilon _{ij}},{\varepsilon _{ij}}\sim N(0,{\sigma ^2})$$

Level 2 (school-level):

$${\beta _{0j}}\,=\,{\gamma _{00}}+{\mu _{0j}},{\mu _{0j}}\sim N(0,{\tau _{00}})$$

Where *Yij* is the IA of student *i* in class *j*; *β*_0*j*_ is the mean IA of class *j*; *γ*_00_ is the grand mean of IA across all classes; *ε*_*ij*_ and *µ*_0*j*_ are the random error of the student and school level, respectively; and *σ*^2^ and *τ*_00_ are the variations at the two levels, respectively.

Table 2 shows the parameter estimation results of the multilevel modeling analysis. According to Cohen, an ICC_1_ lower than 0.059 indicates a weak intra-group correlation, the range from 0.059 to 0.138 suggests a moderate correlation and an ICC_1_ higher than 0.138 indicates a strong correlation. In general, as long as ICC_1_ exceeds 0.059, it is necessary to consider inter-group effects in statistical modeling processing [55]. Meanwhile, ICC_2_ greater than 0.70 indicates good reliability of group mean scores [56]. Model 1 demonstrated that ICC_1_ = 0.269 / (0.269 + 2.635) = 0.093 and ICC_2_ = 0.785. This indicates that about 9.30% of the total variation in IA was caused by differences between classes, which required cross-level data analysis.

#### Results for the model with student-level variables only

The Model 2 is as follows:

Level 1 (student-level):

$$\begin{aligned}{Y_{ij}} = {\beta _{0j}} + {\beta _{1j}}\left( {{\rm{Gender}}} \right) + {\beta _{2j}}\left( {{\rm{Age}}} \right) + {\beta _{3j}}\left( {{\rm{Self-esteem}}} \right) \\ + {\beta _{4j}}\left( {{\rm{Father\,phubbing}}} \right) + {\beta _{5j}}\left( {{\rm{Mother\,phubbing}}} \right) + {\varepsilon _{ij}} \cr \end{aligned}$$

Level 2 (school-level):

$${\beta _{0j}}\,=\,{\gamma _{00}}+{\mu _{0j}}$$

$${\beta _{1j}}\,=\,{\gamma _{10}}+{\mu _{1j}}$$

$${\beta _{2j}}\,=\,{\gamma _{20}}+{\mu _{2j}}$$

$${\beta _{3j}}\,=\,{\gamma _{30}}+{\mu _{3j}}$$

$${\beta _{4j}}\,=\,{\gamma _{40}}+{\mu _{4j}}$$

$${\beta _{5j}}\,=\,{\gamma _{50}}+{\mu _{5j}}$$

From the parameter estimation results of the multilevel modeling analysis presented in Table 2, it was found that student-level variables (i.e., gender, self-esteem, and mother phubbing) had significant predictive effects on students’ IA, except for age and father phubbing. The positive coefficient of gender indicated that boys are more likely than girls to be addicted to the Internet. The positive coefficient of mother phubbing showed that the higher the level of mother phubbing, the higher the probability of children developing IA. Meanwhile, the negative coefficient of self-esteem demonstrated that adolescents with lower levels of self-esteem were more prone to IA.

#### Results for the model with both student- and school-level variables

The final Model 3 is as follows:

Level 1 (student-level):

$$\begin{aligned}{Y_{ij}} = {\beta _{0j}} + {\beta _{1j}}\left( {{\rm{Gender}}} \right)+ {\beta _{2j}}\left( {{\rm{Age}}} \right) + {\beta _{3j}}\left( {{\rm{Self - esteem}}} \right) \\+ {\beta _{4j}}\left( {{\rm{Father\,phubbing}}} \right) + {\beta _{5j}}\left( {{\rm{Mother\,phubbing}}} \right)+ {\varepsilon _{ij}} \cr \end{aligned}$$

Level 2 (school-level):

$$\begin{aligned}{\beta _{0j}} =& {\gamma _{00}} + {\gamma _{01}}\left( {{\rm{Learning\,burden}}} \right) \\&+ {\gamma _{02}}\left( {{\rm{Teacher - student\,relationship}}} \right) + {\mu _{0j}} \cr \end{aligned}$$

$${\beta _{1j}}\,=\,{\gamma _{10}}+{\mu _{1j}}$$

$${\beta _{2j}}\,=\,{\gamma _{20}}+{\mu _{2j}}$$

$$\begin{aligned}{\beta _{3j}}{\kern 1pt} =& {\kern 1pt} {\gamma _{30}}{\kern 1pt}+ {\gamma _{31}}\left( {{\rm{Learning\,burden}}} \right) \\&+ {\gamma _{32}}\left( {{\rm{Teacher - student\,relationship}}} \right) + {\mu _{3j}} \cr \end{aligned}$$

$${\beta _{4j}}\,=\,{\gamma _{40}}+{\mu _{4j}}$$

$$\begin{aligned}{\beta _{5j}}{\mkern 1mu}=& {\mkern 1mu} {\gamma _{50}}{\mkern 1mu}+ {\gamma _{51}}\left( {{\rm{Learning\,burden}}} \right) \\&+ {\gamma _{52}}\left( {{\rm{Teacher - student\,relationship}}} \right) + {\mu _{5j}} \cr \end{aligned}$$

**Fig. 2:**
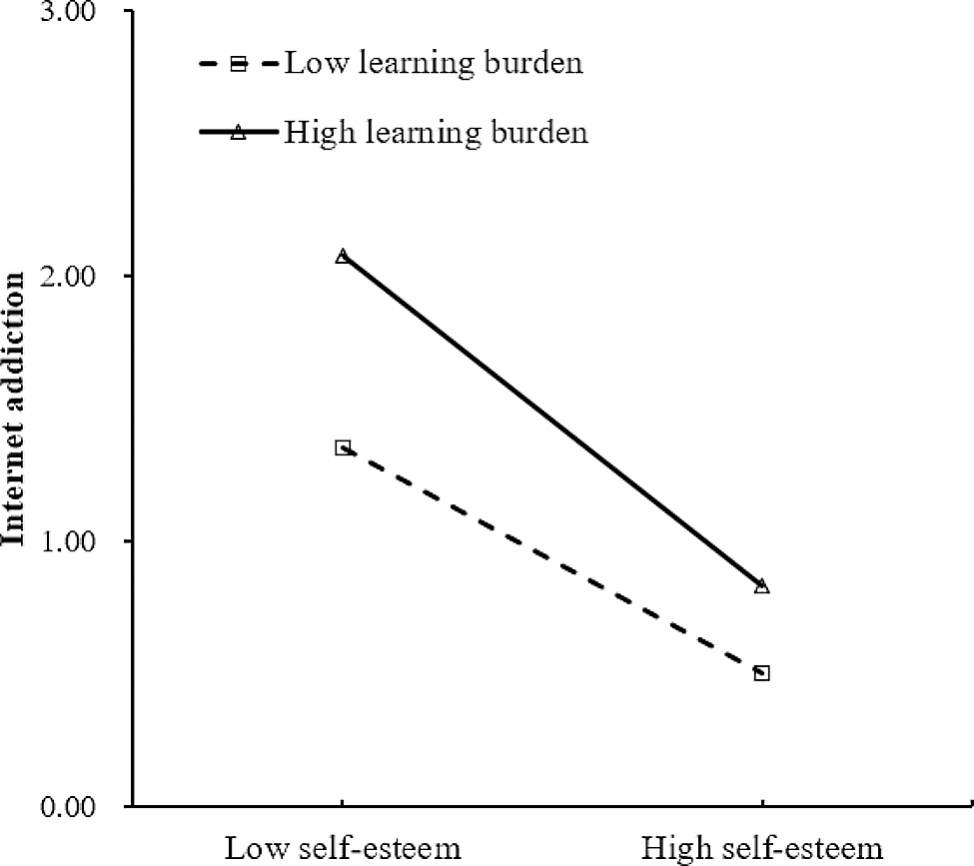
Moderating effect of learning burden in the association between self-esteem and internet addiction

**Fig. 3:**
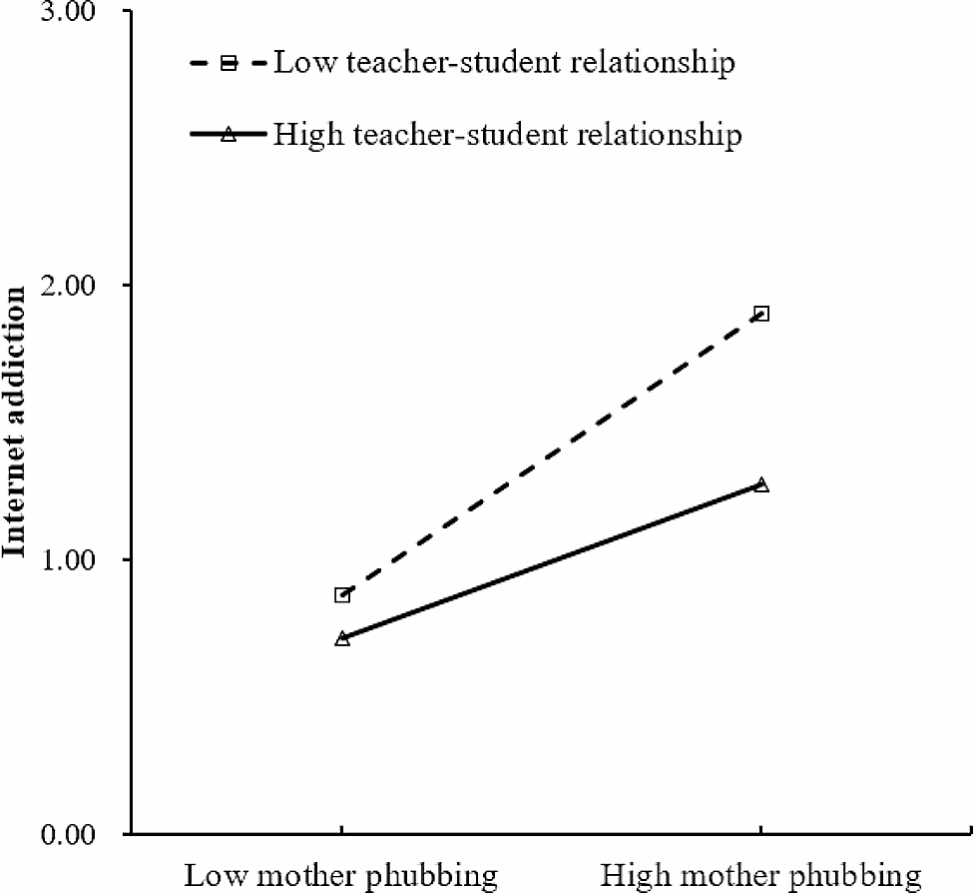
Moderating effect of the teacher-student relationship in the association between mother phubbing and internet addiction

At the school level, it was found that only the learning burden was significantly and positively related to adolescents’ IA. The heavier the classes’ learning burden, the more likely they are to become addicted to the Internet. While the teacher-student relationship had no significant negative relationship with IA. Notably, there were significant interactions between school-level variables and student-level indicators in relationship to IA. First, the relationship between self-esteem and IA was significantly moderated by the learning burden. Second, the teacher-student relationship had a significant moderating effect on the association between mother phubbing and IA. Specifically, the learning burden strengthened the negative relationship between self-esteem and IA, while the teacher-student relationship weakened the positive correlation between mother phubbing and IA (see Table 2). According to Preacher, Curran [57], simple slope tests were adopted and one standard deviation above and below the mean (*M ± 1SD*) represented high and low values for the moderator variable. The significant moderating effects are shown graphically in Figs. 2 and 3.

**Table 2:** The unconditional model and conditional models

Parameter	IA		
	Model 1	Model 2	Model 3
**Fixed effect**			
*Student-level variables*			
Intercept (*γ*_*00*_)	1.197***	1.195***	1.193***
Gender (*γ*_*10*_)		0.277***	0.273***
Age (*γ*_*20*_)		0.028	0.032
Self-esteem (*γ*_*30*_)		−0.078***	−0.077***
Father phubbing (*γ*_*40*_)		0.007	0.008
Mother phubbing (*γ*_*50*_)		0.028**	0.028**
*School-level variables*			
Learning burden (*γ*_*01*_)			0.073*
Teacher-student relationship (*γ*_*02*_)			-0.032
*Interaction between student- and school-level variables*			
Learning burden × Self-esteem (*γ*_*31*_)			−0.007*
Teacher-student relationship × Mother phubbing (*γ*_*52*_)			−0.004**
**Random effect variance component**			
U_0_	0.269***	0.282***	0.214***
R	2.635	2.235	2.231
Deviance	7356.896	7127.746	7157.645

*Notes*: Only variables that have statistically significant interactions are listed

* *p* < 0.05, ** *p* < 0.01, *** *p* < 0.001

## Discussion

Based on Ecological Systems Theory [14], this study aims to understand the effects of multi-level factors (i.e., student and school levels) on adolescent IA by using a HLM approach. The student-level variables include self-esteem, father phubbing, and mother phubbing. Learning burden and teacher-student relationship were included as school-level variables. The findings indicated that both student-level variables and school-level variables have predictive effects on adolescent IA. Meanwhile, school-level variables also moderated the association between student-level variables and IA.

### Student level (level 1) variables and adolescent internet addiction

At the student level, self-esteem was significantly associated with adolescent IA, and H1 was supported. This result is in line with that of a previous study [58], which demonstrated that students with low self-esteem are at higher risk of IA compared to adolescents with high self-esteem. This may be because high self-esteem individuals can evaluate themselves in a relatively objective and positive way and establish higher self-worth in the real world. When faced with stress, they can cope constructively and use the internet appropriately [59]. On the contrary, adolescents with low self-esteem usually have a negative self-concept and typically experience more negative emotions; therefore, when faced with stress, they tend to seek self-affirmation and approval from others on the internet [60]. Previous research has shown that the internet can fill a psychological need that people cannot meet in real life by elevating a sense of accomplishment and worth in the virtual world, which can significantly increase the likelihood of IA among low self-esteem adolescents [19].

We also found that mother phubbing could significantly predict adolescents’ IA, while father phubbing cannot, and thus H2_b_ was supported. Social learning theory suggests that individuals’ social behaviors are obtained by observation and imitation, and when people lack relevant experiences or knowledge, they are inclined to look for alternative experiences from others who are important in their lives [61]. As mothers often take on the role of nurturer and caregiver in the family system, they invest more time in caring for the family and child-rearing, and adolescents may often spend more time with their mothers [31, 62]. This means that mothers, as role models, are more likely to have an intergenerational association with adolescents, and their behaviors may be more easily imitated and internalized by adolescents [61, 63]. Therefore, mother phubbing could have a negative effect on adolescent IA.

In contrast, due to work, the family division of labor, and culture, many Chinese fathers tend to serve as the breadwinners and are responsible for dealing with external issues; thus, they relatively have less time to participate in child-rear and often play more of a supportive role in family education [64]. We speculated that this may be the main explanation for the lack of a direct effect of father phubbing on IA in the present study. That is, H2_a_ was not supported.

Besides, it is worth noting that parent who spends long hours looking down at their mobile phone may not be able to give timely attention and respond to their children’s emotional expressions, resulting in lower quality of parent-child interactions and weaker emotional connections between parents and children [65, 66]. Adolescents without family support are more likely to spend a vast amount of time seeking psychological comfort on the internet, which increases their risk of IA. Therefore, it is necessary to pay attention to the impact of parental phubbing behavior, especially mothers’ phubbing behavior, on adolescent IA.

### School level (level 2) variables and adolescent internet addiction

At the school level, learning burden significantly predicted adolescent IA, and H3 was supported. Classes with a higher learning burden tended to have more coursework and more tests and quizzes than classes with a lower learning burden, and this competition-oriented classroom environment may lead to adolescents gradually losing interest in learning, indulgence in the internet, and the development of IA. In addition, a phenomenon called “Neijuan” has recently emerged in China, which can be seen as a social condition that efforts becoming “inflation”, i.e., people have to invest more effort and time in study or work to compete for limited resources [67], which means that students have to face even greater academic burden in a “Neijuan” environment. Therefore, adolescents are prone to use the internet as a way to release their stress, which further increases their likelihood of developing IA [37].

Moreover, the current study showed that the teacher-student relationship could not predict adolescent IA, thus H4 was not supported. This result may be explained by the buffering effect theory of social support which holds that only when an individual is in a state of stress social support can play a beneficial buffer role, protecting the individual from the damage of stress [68]. In other words, social support is more likely to act as a moderating effect, not as a main effect. In the context of youth social networks in schools, good teacher-student relationships are an important source of social support for students [69]. This implies that the teacher-student relationship may only have a significant protective effect on IA when students experience negative pressure to a certain extent, rather than directly predict IA at the class level.

### Interaction effects between student-level and school-level variables on internet addiction

The interaction between self-esteem and school-level variable was significantly associated with adolescent IA, which supported H5_a_. Students in classes with higher learning burdens were more likely to be negatively affected by low self-esteem when it came to IA than those in classes with lower learning burdens. In China, academic achievement is often regarded as the main means to achieve life goals, and most adolescents experience academic pressure. Especially in recent years, the “Neijuan” phenomenon in education has become more pronounced, which forces young people to be in a further state of high pressure and competition [70]. In this case, individuals with higher self-esteem may view the high learning burden in a positive light at this time and tend to treat learning issues such as school assignments, study materials, and exams as challenges and see themselves as capable of meeting the tasks and demands of learning without escaping to the virtual world [71, 72]. In contrast, low self-esteem adolescents often underestimate their abilities, lack confidence in themselves, and are prone to have negative emotions, which is more likely to lead them to IA in a highly competitive environment [19, 73].

Besides, the interaction between mother phubbing and school-level variable had a significantly related to adolescent IA. Thus, H6_c_ was supported. More specifically, the teacher-student relationship significantly moderated the association between mother phubbing and IA, and a classroom atmosphere with good teacher-student relationships can alleviate the detrimental effect of mother phubbing on IA. Mothers and teachers are often the most important adults in providing comfort, guidance, and support for adolescents [38, 74]. When adolescents are negatively impacted in the family, compared to a class with a poor teacher-student relationship, if they perceive the teacher-student atmosphere in the class as understanding, warm, and caring, then this will provide a safe harbor for adolescents and give them the courage to face their difficulties [74], which may compensate to some extent for the adverse effects of the mother’s phubbing behavior. Therefore, good teacher-student relationships could reduce the likelihood of IA in adolescents from the family environment with high level of mother phubbing behaviors.

## Limitations and implications

Our study has several limitations. First, due to the cross-sectional research design, the findings can only provide predictions of the relationships among variables. Causal relationships between variables and data results from interaction effects should be further tested in a longitudinal follow-up study with repeated measures. Second, all data were collected through self-report questionnaires, and biases in recall and/or social desirability cannot be ruled out as influences on the results. Data could be collected from multiple sources of information (e.g. teacher evaluations, parental reports) to increase its representativeness and reliability in future studies. Third, we found two moderating effects of school-level variables on student-level variables, and the remaining four moderating effects were not significant in this study. Future research is necessary to further investigate the reasons for the non-significant effects and the potential mechanisms that may exist. Fourth, the dependent variable of IA was analyzed as a continuous variable in the present study, which may ignore the information of comparison between different groups. Future studies may consider using clinical samples to divide subjects into IA and normal groups, to further expand and verify the results of this study. Fifth, this study was discussed based on general IA. Previous studies have shown that distinguishing specific subtypes of IA is also a valuable research perspective, as predisposition factors may vary by IA types (e.g., there may be significant gender differences between online game addiction and social media addiction [75, 76]. In addition, to better understand the impact of internet use on individuals, future research needs to include more information, it will be beneficial to take into account what kind of device with what kind of applications in what kind of context is used and how often. Because the use of the internet may also include positive aspects (e.g., online learning, online interpersonal communication), this study only focused on the negative side. Future research needs to combine these different dimensions, which might contribute to getting a more fine-grained understanding of how internet use influences on our lives. Finally, the participants were all adolescent students from China. Considering the role of cultural factors on individual psychology and behavior, the model should be tested within different cultures, including collectivist and individualist societies.

The theoretical and practical implications of this study are as follows. From a theoretical perspective, it examines the factors related to adolescent IA from ecological systems theory based on multilevel data analysis and deepens the understanding of ecological systems theory and the formation mechanism of IA. Specifically, not only do different levels of ecological systems (i.e., individual factors, class factors) have an effect on adolescent IA, but also different ecological systems play a linkage role in adolescent IA (i.e., the interaction between mother phubbing and class teacher-student relationship). From a practical perspective, our study explores the issue from individual, family, and school contexts that may help guide the prevention and intervention of adolescent IA. The findings suggest that a three-pronged approach and collaborative effort could be more effectively used to reduce the risk of IA, namely, increasing adolescent self-esteem levels (individual), decreasing mother phubbing (family), and reducing the learning burden and fostering good teacher-student relationships (school). First, parents and schools can consider fostering children’s self-esteem in daily life and providing training on self-esteem. For example, mindfulness practice is an option to consider. Previous studies have shown that mindfulness exercises can help individuals with low self-esteem focus their attention on the direct experiences they experience, without being overly influenced by negative beliefs or critical thoughts, which has a positive impact on the improvement of their self-esteem [77, 78]. Second, the positive role of a warm and harmonious family environment in the development of adolescents needs to be noted. We encourage parents to develop good smartphone use habits and pay more attention to their children’s psychological needs to improve parent-child communication skills [79]. Furthermore, it is recommended that family therapy be considered when intervening in adolescent IA [80]. Third, schools need to be concerned about the impact of the teacher-student relationship and learning burden on adolescents in the classroom. As important guides on students’ learning paths, teachers should strive to provide a supportive environment for students so that they can grow up in a relaxed and happy classroom atmosphere. At the same time, the education department should formulate relevant policies to address the problem of high schoolwork pressure among adolescents. For example, to reduce the current degree of “Neijuan”, the performance-oriented evaluation system in families and schools should be weakened, and an educational environment suitable for the all-round development of the adolescents should be created, which will help to reduce the problem of adolescent IA.

## Conclusions

In conclusion, this study explored the factors linked to Chinese adolescents’ IA from a multilevel perspective using a HLM. Notably, the relationship between student-level variables and IA can be moderated by class-level variables. The learning burden can exacerbate the probability of low self-esteem adolescents developing IA, while the teacher-student relationship can moderate the adverse effects of mothers’ phubbing behavior on adolescent IA. This study provides a reference value for understanding the mechanism of adolescent IA, thereby supporting the development of targeted interventions to jointly improve the status of adolescent IA from the perspective of individuals, families, and schools.

## Data Availability

The datasets used and analyzed during the current study are not publicly available, as the participants were not asked to consent to publication within repositories but are available from the corresponding author on reasonable request.

## Abbreviations

IA: Internet addiction
DSM-IV: Diagnostic and Statistical Manual of Mental Disorders, 4th Edition
HLM: Hierarchical Linear Modeling
IAD-DQ: Young’s 8-item Diagnostic Questionnaire of Internet Addiction
SD: Standard deviations
ICC: Intra-class correlation coefficients
M ± 1SD: Standard deviation above and below the mean

## References

[1] Young KS (1998). Internet addiction: the emergence of a new clinical disorder. CyberPsychol Behav.

[2] Hall AS, Parsons J (2001). Internet addiction: College student case study using best practices in cognitive behavior therapy. J Mental Health Counseling.

[3] Davis RA (2001). A cognitive-behavioral model of pathological internet use. Comput Hum Behav.

[4] Casaló LV, Escario J-J (2019). Predictors of excessive internet use among adolescents in Spain: the relevance of the relationship between parents and their children. Comput Hum Behav.

[5] Trumello C, Vismara L, Sechi C, Ricciardi P, Marino V, Babore A. Internet addiction: the role of parental care and Mental Health in Adolescence. Int J Environ Res Public Health. 2021;18(24). 10.3390/ijerph182412876 PMC8700934 34948485

[6] Marin MG, Nuñez X, de Almeida RMM (2021). Internet addiction and attention in adolescents: a systematic review. Cyberpsychol Behav Soc Netw.

[7] Wang H, Zhou X, Lu C, Wu J, Deng X, Hong L (2011). Problematic internet use in high school students in Guangdong Province, China. PLoS ONE.

[8] Xu J, Shen LX, Yan CH, Hu H, Yang F, Wang L (2012). Personal characteristics related to the risk of adolescent internet addiction: a survey in Shanghai, China. BMC Public Health.

[9] Cheng Y-S, Tseng P-T, Lin P-Y, Chen T-Y, Stubbs B, Carvalho AF et al. Internet addiction and its relationship with suicidal behaviors: a Meta-analysis of multinational observational studies. J Clin Psychiatry. 2018;79(4). 10.4088/JCP.17r11761 29877640

[10] Lin YJ, Hsiao RC, Liu TL, Yen CF (2020). Bidirectional relationships of psychiatric symptoms with internet addiction in college students: a prospective study. J Formos Med Assoc.

[11] Raj K, Segrave R, Tiego J, Verdéjo-Garcia A, Yücel M (2022). Problematic use of the internet among Australian university students: prevalence and profile. Comput Hum Behav.

[12] Kaess M, Klar J, Kindler J, Parzer P, Brunner R, Carli V (2021). Excessive and pathological internet use - risk-behavior or psychopathology?. Addict Behav.

[13] Shao Y-j, Zheng T, Wang Y-q, Liu L, Chen Y, Yao Y-s (2018). Internet addiction detection rate among college students in the people’s Republic of China: a meta-analysis. Child Adolesc Psychiatry Ment Health.

[14] Bronfenbrenner U. Ecological Models of Human Development: International Encyclopedia of Education, Vol 3, 2nd Ed; 1994.

[15] Throuvala MA, Griffiths MD, Rennoldson M, Kuss DJ (2019). School-based Prevention for adolescent internet addiction: Prevention is the Key. A systematic literature review. Curr Neuropharmacol.

[16] Demo DH (1985). The measurement of self-esteem: Refining our methods. J Pers Soc Psychol.

[17] Rosenberg M. Society and the adolescent self-image. Soc Forces. 1965;3(2).

[18] Chang E, Kim B (2020). School and individual factors on game addiction: a multilevel analysis. Educ Psychol Rev.

[19] Chen HC, Wang JY, Lin YL, Yang SY. Association of Internet Addiction with Family Functionality, Depression, Self-Efficacy and Self-Esteem among early adolescents. Int J Environ Res Public Health. 2020;17(23). 10.3390/ijerph17238820 PMC7731192 33260988

[20] Casale S, Fioravanti G, Bocci Benucci S, Falone A, Ricca V, Rotella F (2022). A meta-analysis on the association between self-esteem and problematic smartphone use. Comput Hum Behav.

[21] Wu CST, Wong HT, Yu KF, Fok KW, Yeung SM, Lam CH (2016). Parenting approaches, family functionality, and internet addiction among Hong Kong adolescents. BMC Pediatr.

[22] Bai Q, Lei L, Hsueh FH, Yu X, Hu H, Wang X (2020). Parent-adolescent congruence in phubbing and adolescents’ depressive symptoms: a moderated polynomial regression with response surface analyses. J Affect Disord.

[23] Liu K, Chen W, Wang H, Geng J, Lei L (2021). Parental phubbing linking to adolescent life satisfaction: the mediating role of relationship satisfaction and the moderating role of attachment styles. Child Care Health Dev.

[24] Zhang Y, Ding Q, Wang Z (2021). Why parental phubbing is at risk for adolescent mobile phone addiction: a serial mediating model. Child Youth Serv Rev.

[25] Nazir T, Bulut S (2019). Phubbing and what could be its determinants: a dugout of literature. Psychology.

[26] Zhou J, Li X, Gong X (2022). Parental phubbing and internet gaming addiction in children: mediating roles of parent-child relationships and depressive symptoms. Cyberpsychol Behav Soc Netw.

[27] Geng J, Lei L, Ouyang M, Nie J, Wang P (2021). The influence of perceived parental phubbing on adolescents’ problematic smartphone use: a two-wave multiple mediation model. Addict Behav.

[28] Zhao J, Ye B, Luo L, Yu L (2022). The effect of parent phubbing on Chinese adolescents’ smartphone addiction during COVID-19 pandemic: testing a Moderated Mediation Model. Psychol Res Behav Manag.

[29] Niu G, Yao L, Wu L, Tian Y, Xu L, Sun X (2020). Parental phubbing and adolescent problematic mobile phone use: the role of parent-child relationship and self-control. Child Youth Serv Rev.

[30] Keizer R, Helmerhorst KOW, van Rijn-van Gelderen L (2019). Perceived Quality of the mother–adolescent and Father–adolescent attachment relationship and adolescents’ self-esteem. J Youth Adolesc.

[31] Renk K, Roberts R, Roddenberry A, Luick M, Hillhouse S, Meehan C (2003). Mothers, fathers, gender role, and Time Parents spend with their children. Sex Roles.

[32] Li D, Zhou Y, Li X, Zhou Z (2016). Perceived school climate and adolescent internet addiction: the mediating role of deviant peer affiliation and the moderating role of effortful control. Comput Hum Behav.

[33] Zhu J, Zhang W, Yu C, Zhou S, Sun G, Zhen S (2015). School Climate and pathological online game use among adolescents:the Moderated Mediation Model. Psychol Dev Educ.

[34] Yang C, Sharkey JD, Reed LA, Chen C, Dowdy E (2018). Bullying victimization and student engagement in elementary, middle, and high schools: moderating role of school climate. Sch Psychol Q.

[35] Wang M-T, Degol JL (2016). School Climate: a review of the Construct, Measurement, and impact on Student outcomes. Educ Psychol Rev.

[36] Li Y, Wang Y, Ren Z, Gao M, Liu Q, Qiu C (2020). The influence of environmental pressure on internet use disorder in adolescents: the potential mediating role of cognitive function. Addict Behav.

[37] Jun S, Choi E (2015). Academic stress and internet addiction from general strain theory framework. Comput Hum Behav.

[38] Wentzel KR. Teacher-student relationships. Handbook of motivation at school. Routledge; 2016. pp. 211–30.

[39] Bowlby J (1982). Attachment and loss: retrospect and prospect. Am J Orthopsychiatr.

[40] Jia J, Li D, Li X, Zhou Y, Wang Y, Sun W (2017). Psychological security and deviant peer affiliation as mediators between teacher-student relationship and adolescent internet addiction. Comput Hum Behav.

[41] Wang P, Gan X, Li H, Jin X (2023). Parental marital conflict and internet gaming disorder among Chinese adolescents: the multiple mediating roles of deviant peer affiliation and teacher-student relationship. PLoS ONE.

[42] Li C, Dang J, Zhang X, Zhang Q, Guo J (2014). Internet addiction among Chinese adolescents: the effect of parental behavior and self-control. Comput Hum Behav.

[43] Wang P, Hu H, Mo PKH, Ouyang M, Geng J, Zeng P (2022). How is Father Phubbing Associated with adolescents’ social networking sites Addiction? Roles of narcissism, need to Belong, and loneliness. J Psychol.

[44] Kweon YR, Park MS (2012). Effects of School Adjustment on higher Grade Elementary School Students’ internet game addiction: focused on gender difference. J Korean Acad Psychiatr Ment Health Nurs.

[45] Rosenberg M. Society and the adolescent self-image. Princeton university press; 2015.

[46] Ding Q, Wang Z-q, Yong-xin Z (2020). Revision of the Chinese version of parents phubbing scale in adolescents. Chin J Clin Psychol.

[47] Jiang G. Class Environment in the Chinese School System: structure and meassurement. Psychol Sci. 2004(04):839–43.

[48] Li Y, Zhong B, Liu X, Zhang Y, Zhu J, Hao W (2012). Reliability and validity of the Chinese version of self-rating Young’s diagnostic questionnaire of internet addiction: a preliminary study. Chin J Drug Depend.

[49] Bryk AS, Raudenbush SW. Hierarchical linear models: applications and data analysis methods. Advanced qualitative techniques in the social sciences. Volume 1. Sage; 2002.

[50] Su A, He W (2020). Exploring factors linked to the mathematics achievement of ethnic minority students in China for Sustainable Development: a multilevel modeling analysis. Sustainability.

[51] Raudenbush SW, Bryk AS. Hierarchical linear models: applications and data analysis methods. sage; 2002.

[52] Hox JJ, Moerbeek M, Van de Schoot R. Multilevel analysis: Techniques and applications: Routledge; 2017.

[53] Bliese PD (2000). Within-group agreement, non-independence, and reliability: implications for data aggregation and analysis. Multilevel theory, research, and methods in organizations: foundations, extensions, and new directions.

[54] Shrout PE, Fleiss JL (1979). Intraclass correlations: uses in assessing rater reliability. Psychol Bull.

[55] Cohen J (2013). Statistical Power Analysis for the behavioral sciences.

[56] LeBreton JM, Senter JL (2007). Answers to 20 questions about Interrater Reliability and Interrater Agreement. Organ Res Methods.

[57] Preacher KJ, Curran PJ, Bauer DJ (2006). Computational tools for probing interactions in multiple Linear regression, Multilevel modeling, and latent curve analysis. J Educ Behav Stat.

[58] Kim H-K, Davis KE (2009). Toward a comprehensive theory of problematic internet use: evaluating the role of self-esteem, anxiety, flow, and the self-rated importance of internet activities. Comput Hum Behav.

[59] Andreassen CS, Pallesen S, Griffiths MD (2017). The relationship between addictive use of social media, narcissism, and self-esteem: findings from a large national survey. Addict Behav.

[60] Peng W, Li D, Li D, Jia J, Wang Y, Sun W (2019). School disconnectedness and adolescent internet addiction: mediation by self-esteem and moderation by emotional intelligence. Comput Hum Behav.

[61] Bandura A, Walters RH. Social learning theory. Englewood cliffs Prentice Hall; 1977.

[62] Lewis C, Lamb ME (2003). Fathers’ influences on children’s development: the evidence from two-parent families. Educ Psychol Rev.

[63] Jamison TB, Lo HY (2021). Exploring parents’ ongoing role in romantic development: insights from young adults. J Soc Pers Relatsh.

[64] Kwok SYCL, Ling CCY, Leung CLK, Li JCM (2013). Fathering Self-Efficacy, marital satisfaction and Father involvement in Hong Kong. J Child Fam Stud.

[65] Hiniker A, Sobel K, Suh H, Sung Y-C, Lee CP, Kientz JA. Texting while Parenting: How Adults Use Mobile Phones while Caring for Children at the Playground. Proceedings of the 33rd Annual ACM Conference on Human Factors in Computing Systems; Seoul, Republic of Korea: Association for Computing Machinery; 2015. p. 727–36.

[66] McDaniel BT, Radesky JS (2018). Technoference: parent distraction with Technology and associations with child behavior problems. Child Dev.

[67] Gu X, Mao EZ (2022). The impacts of academic stress on college students’ problematic smartphone use and internet gaming disorder under the background of neijuan: hierarchical regressions with mediational analysis on escape and coping motives. Front Psychol.

[68] Cohen S, Wills TA (1985). Stress, social support, and the buffering hypothesis. Psychol Bull.

[69] Zhang D, Jin B, Cui Y (2022). Do teacher autonomy support and teacher–student relationships Influence Students’ Depression? A 3-Year longitudinal study. School Ment Health.

[70] Yi D, Wu J, Zhang M, Zeng Q, Wang J, Liang J et al. Does Involution cause anxiety? An empirical study from Chinese universities. Int J Environ Res Public Health. 2022;19(16). 10.3390/ijerph19169826 PMC9408648 36011462

[71] Mulyadi S, Rahardjo W, Basuki AMH (2016). The role of parent-child relationship, Self-esteem, academic self-efficacy to academic stress. Procedia Soc Behav Sci.

[72] Naseri L, Mohamadi J, Sayehmiri K, Azizpoor Y (2015). Perceived Social Support, Self-Esteem, and internet addiction among students of Al-Zahra University, Tehran, Iran. Iran J Psychiatry Behav Sci.

[73] Chu HS, Tak YR, Lee H (2020). Exploring psychosocial factors that influence smartphone dependency among Korean adolescents. PLoS ONE.

[74] Zhen R, Liu R-D, Hong W, Zhou X (2019). How do interpersonal relationships relieve adolescents’ problematic Mobile phone use? The roles of loneliness and motivation to Use Mobile Phones. Int J Environ Res Public Health.

[75] Lopez-Fernandez O. Generalised Versus Specific Internet use-related addiction problems: a mixed methods study on internet, Gaming, and Social networking behaviours. Int J Environ Res Public Health. 2018;15(12). 10.3390/ijerph15122913 PMC6313434 30572652

[76] Su W, Han X, Yu H, Wu Y, Potenza MN (2020). Do men become addicted to internet gaming and women to social media? A meta-analysis examining gender-related differences in specific internet addiction. Comput Hum Behav.

[77] Pepping CA, O’Donovan A, Davis PJ (2013). The positive effects of mindfulness on self-esteem. J Posit Psychol.

[78] Radell ML, Myers CE, Beck KD, Moustafa AA, Allen MT (2016). The personality trait of intolerance to uncertainty affects behavior in a Novel Computer-based conditioned Place Preference Task. Front Psychol.

[79] Mi Z, Cao W, Diao W, Wu M, Fang X (2023). The relationship between parental phubbing and mobile phone addiction in junior high school students: a moderated mediation model. Front Psychol.

[80] Zajac K, Ginley MK, Chang R, Petry NM (2017). Treatments for internet gaming disorder and internet addiction: a systematic review. Psychol Addict Behav.

